# Development and clinical implementation of a care guideline to support self-management and responsibility transition in adolescents and young adults with biliary atresia

**DOI:** 10.1016/j.hctj.2026.100150

**Published:** 2026-07-28

**Authors:** Katsuhiro Hiratsuka, Hiroko Watayo, Nobue Nakamura

**Affiliations:** a Department of Nursing, School of Nursing and Rehabilitation Sciences, Showa Medical University, Kanagawa, Japan; b Department of Pediatric General and Urogenital Surgery, Juntendo University School of Medicine, Tokyo, Japan; c Department of Nursing Science, Uekusa Gakuen University, Chiba, Japan

**Keywords:** Adolescent and young adult, Responsibility transition, Biliary atresia, Care guideline, Clinical implementation

## Abstract

**Objective:**

This study developed, refined, and implemented a care guideline for adolescents and young adults (AYAs) with biliary atresia surviving with their native livers and their parents, based on a responsibility transition process model. We evaluated its clinical usefulness and identified challenges related to sustainable implementation. This study extends a previously developed responsibility transition model by translating it into a clinically implemented guideline and examining its real-world applicability.

**Methods:**

An implementation-focused, multiphase mixed-methods design was used. Phase 1 involved drafting a care guideline addressing developmental stages, daily life adjustment, parental role changes, and potential future liver transplantation. Phase 2 comprised expert reviews by five pediatric nursing professionals. In Phase 3, three experienced outpatient nurses implemented the guideline with six AYA–parent dyads (AYAs aged 15–22 years) over 12 months. Nurse group interviews were qualitatively analyzed, and transition readiness was assessed before and after the intervention.

**Results**:**

Qualitative analysis identified four categories, revealing usefulness and challenges as intertwined in daily practice. Descriptive questionnaire findings showed relatively stable knowledge scores, whereas behavior scores tended to decline among both AYAs and parents over the 12-month period. Guideline usefulness was perceived as a dynamic implementation process shaped by facilitating and constraining factors operating at individual, team, and organizational levels.

**Conclusion:**

This study translated a responsibility transition process model into an implementable care guideline and demonstrated its feasibility within routine pediatric outpatient care. Contextual barriers to sustained use, including workload, role clarity, and organizational integration, were identified, providing directions for future refinement and broader application to AYAs with other childhood-onset chronic conditions.

## Highlights

• A care guideline supported responsibility transition in AYAs with biliary atresia.
• Clinical implementation demonstrated feasibility in routine outpatient care.
• Relational, team, and organizational factors shaped guideline use.
• Contextual barriers to sustained implementation were identified.

## Graphical Abstract

**Figure:**
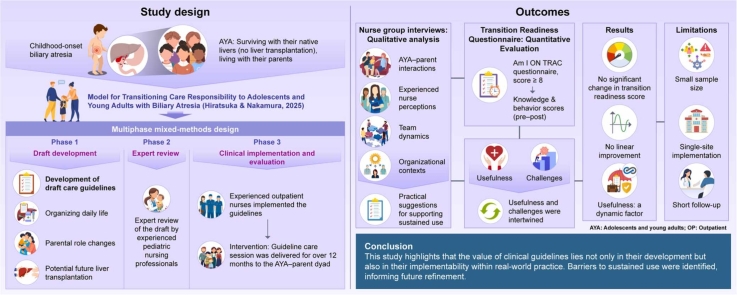

## 1 Introduction

Advances in medical care have markedly improved the survival rates of individuals with chronic childhood-onset conditions. As increasing numbers of individuals reach adolescence and young adulthood, attention has shifted toward supporting the transition to adult roles and enhancing self-management. Transition preparation should not be determined solely by chronological age but should reflect developmental stage and readiness. Established health care transition frameworks recommend initiating structured preparation in early adolescence and promoting the gradual transfer of care responsibility through shared decision-making and individual readiness.^1^ Contemporary definitions of health care transition define it as the process of moving from a child-centered model of care to an adult-centered model of care, with or without transfer to a new clinician. This broader perspective highlights the importance of supporting responsibility transfer, self-management, and evolving parent–child roles even among AYAs who continue receiving care within pediatric settings. Barriers to health care transition have been identified at multiple levels, including insufficient preparation for self-management, parental difficulty relinquishing responsibility, and healthcare-system challenges such as inadequate communication, limited provider training, and insufficient coordination across services.^1^

Support for adolescents and young adults (AYAs) with childhood-onset chronic conditions during the transition period requires attention to their developmental stage and the promotion of autonomy in self-management. Developmentally appropriate care supports AYAs by gradually assuming responsibility for their health management.^2^ Concurrently, parental involvement remains an important factor in the development of autonomy and self-care skills. Empirical studies indicate that during the transition toward self-management, parents often function both as monitors and supportive partners while maintaining collaborative relationships with their children.^3^ Parental and caregiver involvement has also been associated with higher self-efficacy, greater autonomy, and more proactive communication with healthcare professionals.^4^ Lee et al. demonstrated that competence and autonomy develop progressively through a feedback loop in which successful self-management increases parental trust,^5^ thereby facilitating the gradual transfer of responsibility to more complex tasks.

Biliary atresia is a progressive fibroinflammatory disease of the biliary system and a leading cause of neonatal cholestasis. Although Kasai portoenterostomy is performed as the initial treatment, approximately 60–75% of patients require liver transplantation by 18 years of age.^6^ Therefore, AYAs with biliary atresia surviving with their native livers face ongoing uncertainty regarding the potential need for future transplantation. Such uncertainty can influence both quality of life and the development of self-management abilities.^7^ In Japan, approximately 95% of living-donor liver transplants are provided by parents.^8^ This context creates a distinctive relational dynamic: parents may hesitate to transfer caregiving responsibilities to their children and may experience conflict between their ongoing caregiving role and the possibility of becoming future living donors.^9^ Consequently, transition support in AYAs with biliary atresia surviving with their native livers must address not only AYAs’ readiness but also parental psychological states and evolving parent–child relationships. This population was specifically targeted because uncertainty regarding the timing and necessity of future living-donor liver transplantation may persist for many years and may shape distinctive developmental and relational trajectories.

A previously developed process model of responsibility transition in AYAs with biliary atresia conceptualizes these relational dynamics.^10^ This model provides a framework for understanding relational changes and responsibility transfer between parents and AYAs. However, theoretical frameworks may not necessarily be translated into routine clinical practice without concrete tools that guide assessment, intervention timing, and the application of phase-specific characteristics to everyday nursing care. Consequently, theoretical insights may remain underutilized. Moreover, as argued by Gagliardi et al.,^11^ the value of clinical guidelines lies not only in their development but also in their implementability—that is, the extent to which they are usable, adaptable, and applicable within real-world clinical contexts. Therefore, in healthcare transition research, it is essential not only to articulate theoretical models but also to translate them into practical tools and to examine their performance through social implementation.

Accordingly, the present study developed a care guideline for AYAs with biliary atresia surviving with their native livers, as well as for their parents. The guideline was designed to support AYAs’ independence and autonomy in organizing their daily lives with illness, facilitate the transfer of care responsibility from parents to AYAs, and guide healthcare professionals, particularly nurses, in responding to challenges emerging within this process. Furthermore, the guideline was implemented in clinical settings to examine its usefulness and identify the challenges revealed through the implementation process. By focusing on both developmental and real-world applications, this study aims to bridge the gap between process models and practice.

This study aimed to develop an initial care guideline, based on a previously developed process model of responsibility transition, for AYAs with biliary atresia surviving with their native livers, as well as for their parents, and to refine it through expert review by experienced pediatric nursing professionals. In addition, this study evaluated its clinical usefulness and explored challenges related to sustainable implementation in routine clinical settings.

## 2 Methods

### 2.1 Study design

This study was designed as an implementation-focused, multiphase, mixed-methods study rather than as an effectiveness trial. The aim was to develop, implement, and examine a care guideline to support AYAs with biliary atresia surviving with their native livers in organizing their daily lives while managing their illness. A mixed-methods approach was adopted to capture relational and process-oriented changes not fully reflected in quantitative indicators alone and to enable the integration of qualitative and quantitative findings.

This study comprised three phases:

Phase 1: Development of a draft care guideline (2018).

Phase 2: Refinement of the draft guideline through expert review (2018).

Phase 3: Clinical implementation of the guideline and examination of its usefulness and challenges (2023–2024).

In Phases 1 and 2, the content of the care guideline was developed and refined based on prior research findings related to transition support for AYAs with biliary atresia surviving with their native livers, as well as their parents. In Phase 3, the finalized guideline was implemented in clinical practice, and its usefulness and challenges for sustained application were examined. The draft guideline was initially developed in 2018 based on a theoretically derived model emphasizing parent–child relational dynamics. It was subsequently implemented and examined in clinical practice during 2023–2024, allowing for assessment of its contemporary applicability.

This study was conducted in accordance with the Standards for Reporting Qualitative Research (SRQR) guidelines.^12^ Reporting of implementation processes and contextual factors was informed by the Standards for Reporting Implementation Studies (StaRI) framework.^13^

### 2.2 Overview of the process model for transitioning care responsibility

**Fig. 1:**
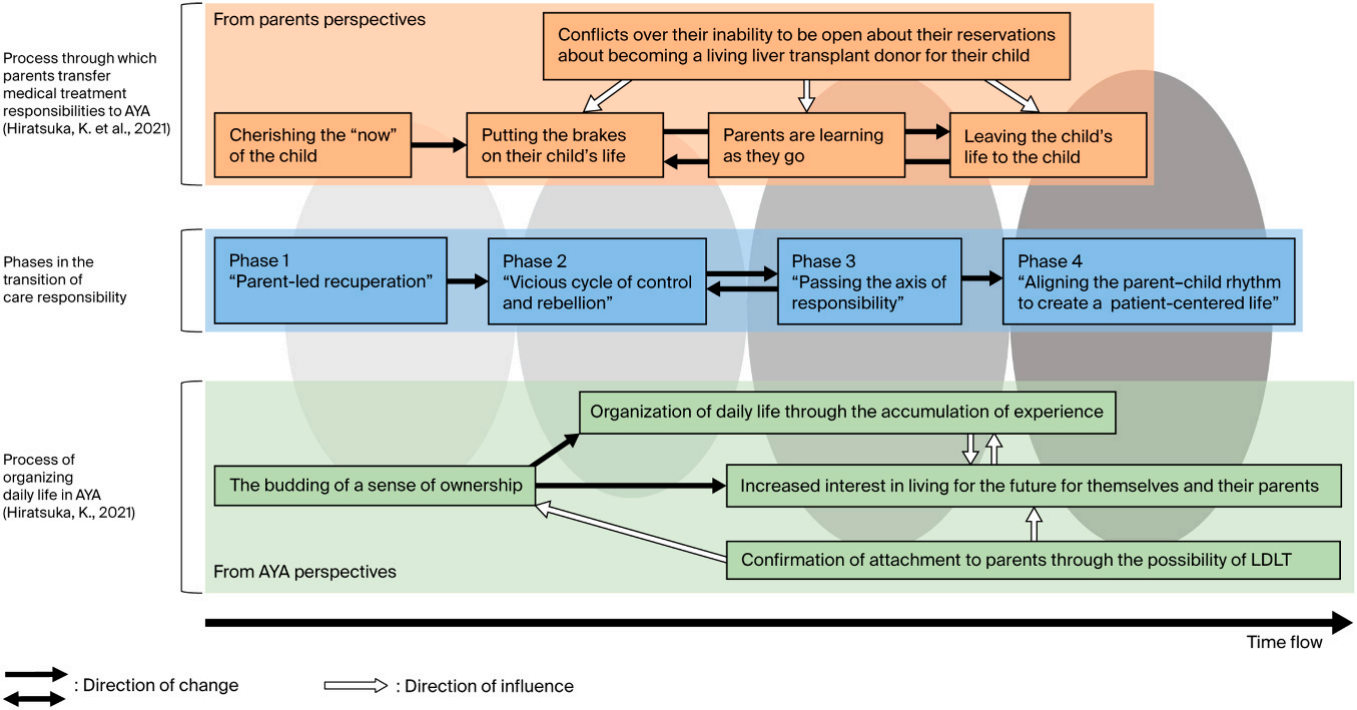
Process model for the transition of daily responsibility from parents to adolescents and young adults with biliary atresia, The model depicts four phases and the shifting roles of AYAs and their parents throughout the transition process. This figure illustrates four phases in the transition of daily responsibility from parents to AYAs with biliary atresia surviving with their native livers. The arrows indicate the direction of change and influence over time. Ovals represent key categories from prior AYA- and parent-focused studies (Hiratsuka, 2021; Hiratsuka et al., 2021), mapped onto the phases as a joint display integrating both perspectives. Reproduced from Hiratsuka, K.; Nakamura, N. (2025). *Process model for transitioning care responsibility to adolescents and young adults with biliary atresia: A secondary and integrative analysis. Nursing Reports, 15*(8), 308. https://doi.org/10.3390/nursrep15080308. Licensed under CC BY 4.0.

The Process Model for Transitioning Care Responsibility to AYAs with Biliary Atresia **(**Fig. 1**)** was previously developed and reported.^10^ Fig. 1 is reproduced under the CC BY 4.0 license to provide the theoretical foundation for the present study. Data were reanalyzed using the modified grounded theory approach, focusing on dyadic parent–child interactions and integrating findings from two prior qualitative studies to derive four phases of responsibility transition.

The four phases of the process model are as follows:

1. Parent-led recuperation;.

2. A vicious cycle of control and rebellion;.

3. Passing the axis of responsibility; and.

4. Aligning the parent–child rhythm to create a patient-centered life.

In this model, “a vicious cycle of control and rebellion” refers to a reciprocal pattern in which parents’ heightened efforts to maintain control over illness management intensify the AYA’s resistance, while the AYA’s resistance further heightens parental concern and control.^10^

The model conceptualizes responsibility transition as a dynamic and context-sensitive process characterized by mutual adaptation between AYAs and parents through communication, negotiation, and reflection. Progression is nonlinear, and movement between phases may occur according to developmental, relational, and clinical circumstances. The model provides the theoretical foundation for developing and implementing the present care guideline.

### 2.3 Key definitions

*AYAs with Biliary Atresia Surviving with their Native Livers*.

In this study, AYAs with biliary atresia surviving with their native livers were defined as individuals diagnosed with biliary atresia who underwent Kasai portoenterostomy as the primary treatment and did not undergo liver transplantation. The AYA period was broadly defined as approximately twelve years of age through the twenties, reflecting developmental and social transitions rather than strict chronological boundaries.

*Daily Life with Illness/Organizing Daily Life with Illness*.

Daily life with illness refers to everyday activities, including school, work, and social interactions, which are influenced by biliary atresia and its treatment and management, and shaped through interactions with family members and others in the surrounding environment.

Organizing daily life during illness extends beyond merely executing specific health-related behaviors. It refers to the ongoing process of managing, coordinating, and adjusting illness-related matters in daily life, including decision-making and prioritization within broader life contexts.

*Responsibility transition*.

Responsibility transition is defined as the gradual shift in responsibility for daily life management, illness management, and health-related decision-making from parents to AYAs. Responsibility transition is understood as a dynamic and context-sensitive process through which AYAs and parents mutually adapt through communication, negotiation, and reflection. Progression is nonlinear, and movement between phases may occur in either direction according to developmental, relational, and clinical circumstances.

### 2.4 Phase 1: Development of the draft care guideline

#### 2.4.1 Aim

The aim of Phase 1 was to develop a draft care guideline for AYAs with biliary atresia surviving with their native livers, as well as their parents, designed to support the process by which AYAs organize their daily lives with illness.

#### 2.4.2 Procedure

The draft care guideline was developed using the Process Model for Transitioning Care Responsibility to AYAs with Biliary Atresia **(**Fig. 1**)** as the primary theoretical framework. In addition, domestic and international literature on healthcare transition, self-management support for individuals with chronic conditions, and parental involvement was reviewed. Representative references included studies on biliary atresia transition and self-management support, long-term outcomes among native liver survivors, and parental involvement in transplantation-related care.^14^, ^15^, ^16^, ^17^, ^18^ The disease-specific characteristics of biliary atresia and the context of living-donor liver transplantation in Japan were incorporated into the guideline content.

The structure of the draft guideline was organized from the following perspectives:

- Developmental stage and life context of AYAs,
- Organizing daily life with illness (medication management, symptom monitoring, and life adjustments),
- Parental involvement and changes in parental roles, and
- Coping with uncertainty, including the potential need for future liver transplantation.

Based on these perspectives, the draft guideline was designed as a practical tool for use in clinical settings. Table 1 presents the components of the draft care guideline and their underlying rationale.

**Table 1:** Components of the draft care guideline and their description and rationale.

Component	Description and rationale
Purpose of the Care Guideline	Clarifies the overall aim of the care guideline, including the target population, intended users (nurses), and the appropriate timing for implementation. This component was developed by integrating participant characteristics, findings from the prior process model study, related transition research, and a review of relevant literature.
Assessment of Physical and Daily Living Status	Presents assessment items and indicators related to patients’ medical conditions and self-management in daily life, complementing the findings from our previous research and relevant transition literature. These items were derived from medical literature and reviews of transition-related studies.
Assessment of Organizing Daily Life with Illness	Provides an assessment framework focusing on patients, parents, and parent–child interactions, aimed at supporting AYAs in organizing their daily lives with illness. This framework was derived from categories, subcategories, and concepts constituting the three core processes identified in our prior studies (Hiratsuka & Nakamura, 2025).
Goals and Nursing Care Strategies	Specifies goals and corresponding nursing care strategies for patients, parents, and parent–child interactions, aiming to support AYAs in organizing their daily lives with illness. These elements were derived through the integration of our prior findings, literature review, and the researchers’ professional experience as pediatric nursing specialists.

### 2.5 Phase 2: Refinement of the draft care guideline

#### 2.5.1 Aim

The aim of Phase 2 was to enhance the clinical validity and usability of the draft care guideline through structured expert review.

#### 2.5.2 Procedure

Phase 2 involved an expert review of the draft care guideline by nursing professionals. Eligible reviewers were nursing professionals with at least five years of clinical experience and substantial experience in caring for AYAs with biliary atresia and their parents. Reviewers evaluated the draft guideline for clinical validity, content clarity, feasibility in outpatient settings, and practical applicability.

#### 2.5.3 Data collection

The expert feedback was collected through semi-structured interviews conducted by the primary researcher. The interviews explored the comprehensibility of the guideline content, its applicability in routine clinical practice, and areas requiring clarification or modification. All interviews were audio-recorded and transcribed verbatim.

#### 2.5.4 Analysis

The interview data were analyzed using qualitative content analysis. Suggestions and identified issues were systematically extracted and categorized. Based on these findings, revisions were made to refine the wording, reorganize the structural components, and enhance usability in clinical settings, resulting in the finalized care guideline.

### 2.6 Phase 3: Clinical implementation and examination of usefulness and challenges

#### 2.6.1 Aim

Phase 3 aimed to implement the finalized care guideline in outpatient clinical practice and to examine its usefulness and the challenges related to sustained use.

#### 2.6.2 Participants

Participants comprised outpatient nurses who implemented the guideline with AYAs with biliary atresia surviving with their native livers, as well as with their parents.

Eligible nurses were outpatient nurses with at least five years of clinical experience and substantial experience caring for AYAs with biliary atresia and their parents. Participants were required to hold positions that allowed them to provide direct care to eligible AYAs and their parents during the study period.

Eligible AYAs and parents were selected according to the eligibility criteria specified in the Intervention section.

#### 2.6.3 Intervention

##### 2.6.3.1 Purpose of this intervention

Based on the Process Model for Transitioning Care Responsibility, this care guideline was designed to support AYAs in organizing and sustaining self-management; enhance psychological and practical preparedness for living with the possibility of future living-donor liver transplantation, and provide healthcare professionals with a structured framework aligned with the model.

The guideline consisted of three core components: an assessment framework based on four domains, phase-specific achievement goals, and phase-specific support strategies designed to facilitate responsibility transition.

##### 2.6.3.2 Delivery of guideline-based care

The intervention was based on the finalized care guideline developed and refined through Phase 2. The core operational components of the guideline used during the implementation period are summarized in **Appendix 1 (**Supplementary Material**)**.

During each encounter, nurses used the guideline to conduct structured assessments across four domains addressing the AYA’s health and daily life, self-management, parental transfer of responsibility, and parent–AYA interactions. Based on these assessments, nurses provisionally identified the responsibility-transition phase, selected phase-specific achievement goals, and implemented individualized support strategies. The four phases derived from the Process Model for Transitioning Care Responsibility included: 1. Parent-led recuperation; 2. A vicious cycle of control and rebellion; 3. Passing the axis of responsibility; and 4. Aligning the parent–child rhythm to create a patient-centered life. These phases represented progressive changes in AYA autonomy and parental involvement.

The primary intervention approach consisted of repeated interviews and structured assessments conducted during these encounters; both AYAs and their parents generally participated in these sessions. Through these repeated encounters, nurses explored AYAs’ self-management, facilitated discussions regarding responsibility transition, supported reflection on parent–AYA interactions, and implemented phase-specific support strategies. Phase identification remained provisional and was revised as needed throughout the implementation period. While the content and focus of each session followed the structure and principles of the guideline, they were individualized according to the circumstances of the AYAs and their parents.

##### 2.6.3.3 Enrollment

The care guideline was originally developed for AYAs with biliary atresia surviving with their native livers, generally aged 12–29 years, who were not experiencing decompensated cirrhosis requiring immediate liver transplantation, as well as for parents or primary caregivers who had played a central role in disease management and accompanying outpatient visits.

For this pilot implementation study, eligible AYAs were provisionally restricted to those aged 15–29 years at initiation of care, surviving with their native livers, and considered clinically appropriate for participation by both the responsible outpatient nurse and attending physician. Clinical appropriateness was determined based on medical stability and the absence of severe psychosocial difficulties, such as depression or acute psychological distress, that might interfere with participation in guideline-based transition support.

AYAs with decompensated cirrhosis for whom liver transplantation had already been determined, as well as those with psychiatric disorders, intellectual disabilities, or other conditions requiring substantially different approaches to care, were excluded. Parents were defined as primary caregivers who had played a central role in the upbringing and medical care of the AYAs.

Potentially eligible AYAs and their parents were approached by the responsible nurse or attending physician during routine outpatient visits. Written and verbal explanations regarding participation in guideline-based care and associated evaluation procedures were provided, and written informed consent was obtained from all participants before enrollment. As this study represented a pilot implementation conducted within routine clinical practice, blinding procedures were not applied.

##### 2.6.3.4 Duration and session frequency

The care guideline was not designed to be implemented within a predefined intervention period, as responsibility transition was conceptualized as a gradual and nonlinear process that may unfold over several years depending on developmental, relational, and clinical circumstances.

For the purposes of this pilot implementation study, participants were followed for 12 months from the first guideline-based outpatient encounter. Guideline-based care was delivered according to usual outpatient follow-up schedules, which typically occurred every few months in clinically stable cases. Details regarding actual implementation fidelity are presented in the Results section.

#### 2.6.4 Evaluation

##### 2.6.4.1 Qualitative evaluation

After the 12-month intervention period, semi-structured group interviews were conducted with outpatient nurses. To capture both positive and negative aspects of implementation, the interview guide included 10 main prompts across four topic areas: (1) perceived usefulness of the care guideline in practice, (2) challenges encountered in sustaining its use, (3) care providers’ characteristics, and (4) other general comments. Questions concerning participants’ characteristics were primarily structured, whereas those exploring their experiences and perceptions were open-ended. Follow-up questions and probes were used flexibly to explore participants’ responses in greater depth. For example, participants were asked, “Could you describe any changes, if any, in the care you provided after using the care guideline?”.

The interviews were audio-recorded, transcribed verbatim, and analyzed inductively using qualitative methods. The qualitative data credibility was enhanced through discussions among multiple researchers, with attention paid not only to positive outcomes but also to identifying barriers, unintended effects, and areas requiring revision.

##### 2.6.4.2 Quantitative evaluation

Transition readiness was assessed before and after the intervention using the Am I ON TRAC for Adult Care Questionnaire (validated Japanese version), which includes both youth and parent versions. The questionnaires were originally developed by Saewyc and the BC Children’s Hospital ON TRAC Transition Initiative^19^, ^20^ as part of the ON TRAC transition program. The Japanese version was translated and psychometrically evaluated in 2019 and demonstrated good internal consistency (Cronbach’s α = 0.84). ^21^

The youth version^19^ comprises 24 items across two domains: knowledge (15 items, four-point scale) and behavior (9 items, five-point scale). Higher scores indicate greater readiness for transition. The behavior subscale includes a cut-off score, with items dichotomized according to predefined criteria; a total score of eight or higher indicates that readiness has been achieved.

The parent version^20^ parallels the youth version and assesses parents’ perceptions of their children’s knowledge and self-management behaviors in preparation for adult care.

In this study, the questionnaire was not used to determine intervention efficacy. Rather, it was employed as a reference indicator to reflect the changes observed during implementation.

Qualitative and quantitative data were integrated during the interpretation. Qualitative findings were used to contextualize and explain the quantitative results related to transition readiness, thereby enabling a comprehensive understanding of the implementation process.

### 2.7 Ethical considerations

This study was conducted in accordance with the Declaration of Helsinki and was approved by the Ethics Committee of the Graduate School of Nursing, Chiba University, Japan (No. 30-17); the Ethics Committee for Research Involving Human Subjects, Sophia University (No. 2020-91); and the Ethics Committee of the Faculty of Medicine, Juntendo University (No. E21-0314).

## 3 Results

This section presents the findings of Phase 2, in which the draft care guideline was refined through expert review, and Phase 3, in which the finalized guideline was implemented in clinical practice to examine its usefulness and the challenges related to continued use.

### 3.1 Phase 2: Refinement of the draft care guideline

#### 3.1.1 Participant characteristics

In Phase 2, expert feedback on the draft care guideline was obtained from five licensed nursing professionals with 5–14 years of experience in caring for AYAs with biliary atresia and their parents. All were registered nurses, including one certified transplant coordinator accredited by the Japan Society for Transplantation, and two held supervisory roles. Three had experience in pediatric surgery or transplant outpatient clinics. Participants were affiliated with urban general or pediatric specialty hospitals that provided surgical treatment for biliary atresia, including liver transplantation.

#### 3.1.2 Overview of expert feedback

The expert interviews yielded feedback that included both confirmation of the guideline’s clinical relevance and suggestions for modifications to enhance its clarity and feasibility in routine practice.

The participants highlighted the importance of aligning nursing support with AYAs’ and parents’ perceptions of, and coping strategies in, daily life—particularly in the context of ongoing uncertainty regarding the potential need for liver transplantation. They described the guideline as a structured reference for assessing parent–child dynamics and planning care across different stages of responsibility transition.

Simultaneously, several areas requiring refinement were identified. Some expressions and structural components were considered abstract or insufficient for routine outpatient use. In particular, participants emphasized the need to clarify (1) the timing of nursing interventions, (2) focal points for supporting both AYAs and parents, and (3) practical considerations for enhancing usability within the time constraints of outpatient settings.

#### 3.1.3 Revisions to the care guideline

Based on the feedback obtained in Phase 2, several revisions were made to the draft care guideline.

First, the support content was reorganized according to developmental stages, and the perspectives guiding nursing interventions were clarified. Second, changes in the roles and involvement of AYAs and parents were illustrated using concrete behavioral examples to facilitate practical understanding. Third, the assessment and documentation components were simplified and reorganized to enhance their usability in routine clinical settings.

Following iterative revisions based on expert review and feasibility testing, the care guideline was finalized for clinical implementation and subsequently used during Phase 3 implementation.

### 3.2 Phase 3: Clinical implementation of the care guideline

#### 3.2.1 Participants and overview of the intervention

In Phase 3, nursing care based on the finalized care guideline was delivered by three outpatient nurses to six AYAs with biliary atresia surviving with their native livers, as well as to their parents (12 individuals in total). The characteristics of the participating AYAs and their parents are summarized in Table 2.

**Table 2:** Background characteristics of adolescents and young adults with biliary atresia surviving with their native livers.

Case	Age (years)	Sex	Educational/work status	Diagnosis of complications (including past history)	Parent’s age (decade)
1	15	Male	Lower secondary school student	Cholangitis	Mother (40 s)
2	18	Male	Upper secondary school student	Cholangitis; esophageal variceal bleeding	Mother (40 s)
3	19	Male	University student	Bowel obstruction (ileus)	Mother (30 s)
4	20	Female	University student	None	Mother (40 s)
5	22	Female	Employed (part-time)	Hypersplenism → partial splenic embolization	Mother (50 s)
6	22	Female	University student	Bowel obstruction (ileus); cholangitis	Mother (40 s)

None of the nurses involved in Phase 3 had participated in Phase 2. Their clinical experience ranged from 8 to 20 years. All patients met the predefined eligibility criteria described in the Methods section. One nurse had completed graduate-level education.

The six participating AYAs ranged in age from 15 to 22 years and included three males and three females. Their educational and employment statuses ranged from secondary school students to university students and working adults. All patients met the eligibility criteria defined in the Methods section and were considered clinically appropriate for participation by both the responsible nurse and the attending physician at the initiation of care. All AYAs had a history of biliary atresia–related complications, including cholangitis, ileus, esophageal variceal bleeding, and hypersplenism requiring partial splenic embolization. However, none had restrictions on their daily activities at the time of the intervention. Five of the six AYAs were receiving ongoing oral medication therapy. One AYA was referred to a transplant center in anticipation of possible future liver transplantation (Case No. 5).

In all cases, the primary caregiver accompanying the AYAs to the outpatient visits and responding to the questionnaire was the mother, whose age ranged from her 30 s to her 50 s. Although parental gender was not specified as an inclusion criterion, this finding may reflect the tendency for mothers to assume primary caregiving responsibilities for children and adolescents with chronic conditions in Japan. However, the small sample size precludes broader generalization.

#### 3.2.2 Implementation fidelity

Each AYA–parent dyad received 5–12 guideline-based sessions over the 12-month implementation period. The dyad receiving 12 sessions attended monthly outpatient visits because of clinical instability. Two hospitalizations for cholangitis occurred during follow-up, and guideline-based interventions were not implemented during inpatient care (Case No. 1).

Sessions were defined as encounters in which guideline-based interviews or structured outpatient nursing documentation were completed. Telephone-only contacts, brief encounters without structured assessment, and consultations conducted exclusively by healthcare professionals who did not utilize the guideline were not considered intervention sessions because they did not provide sufficient opportunity for guideline-based assessment and intervention across the four domains.

No guideline-based interventions were discontinued during the implementation period, and no participants requested withdrawal. In two cases, reduced attendance at scheduled outpatient visits toward the end of follow-up resulted in missing follow-up questionnaires.

To support continuity and monitoring of implementation, a dedicated outpatient file was maintained for participating AYAs throughout the implementation period, and guideline-related documentation was updated longitudinally. Implementation was supported by an initial orientation provided by the principal researcher and by consultation through meetings and email as needed. However, routine application of the guideline and clinical decision-making remained the responsibility of the outpatient nurses.

#### 3.2.3 Implementation experiences of outpatient nurses: usefulness and contextual constraints

Analysis of post-intervention group interviews with outpatient nurses generated four categories capturing implementation experiences: how the guideline shaped nursing assessments and interactions, and which contextual conditions enabled or hindered its sustained use. Four categories were generated from the qualitative analysis, each reflecting both enabling and constraining dimensions of implementation. These categories are presented below with illustrative examples from the nurses’ accounts.

##### 3.2.3.1 Category 1. Facilitating AYAs’ adjustment to daily life while navigating uncertainty about liver transplantation and parent–child dynamics

Use of the guideline helped nurses structure their assessments and conversations, creating opportunities for AYAs to reflect on daily routines and physical conditions, and to make concrete, small-scale adjustments in day-to-day self-management. Concurrently, nurses stated that uncertainty about future liver transplantation and long-standing parent–child dynamics often made it difficult to move beyond immediate adjustments, and sensitive topics were sometimes deferred. The nurses also described encountering both facilitators and constraints simultaneously when providing transition-related care in outpatient settings.

For example, nurses noted that the guideline made it easier to identify entry points for discussing daily life adjustment and assuming greater responsibility for self-management, and that change was more likely when conditions were aligned, such as the timing of life events (e.g., school progression), AYA readiness, and parents’ understanding.

*“It might be good if they can do this before entering high school… by the time they graduate from junior high school, perhaps they could try managing their own medications. Then we could work together toward that goal.” (Nurse C)*.

Another nurse noted that *“the timing happened to be good… graduating from school and preparing for employment. We were able to encourage and acknowledge those efforts.”* (Nurse B).

In contrast, they described difficulties in initiating anticipatory conversations about potential transplantation, including family implications in living-donor transplantation. They also noted the challenge of intervening in parent–child relationships during adolescence and young adulthood when AYAs may be reluctant to accept support that explicitly addresses the parent–child relationship, which could limit the depth and timing of nursing interventions.

*“Regarding lifestyle adjustment, I think I was able to get involved quite a bit. However, when it came to living-donor liver transplantation, there were aspects I simply could not bring myself to discuss.”* (Nurse A).

##### 3.2.3.2 Category 2. Nurses’ shifts in perception of transition support under time and workload constraints

After the implementation of the guideline, nurses described a shift in how they viewed their roles in transition support. They reported recognizing the importance of engaging more proactively with AYAs and their families in routine outpatient care and of sharing relevant observations with physicians and other staff members. Simultaneously, the high volume of pediatric patients in general outpatient settings, together with time constraints and workload demands, was described as a barrier limiting the sustained use of the guideline.

For example, nurses reflected that although they had previously recognized the need to intervene with AYAs in the transition phase, they had often been *“carried along by routine tasks.”* The guideline prompted them to participate more actively in medical consultations and highlighted the importance of collaboration with physicians and other staff members.

Conversely, despite perceiving the need for intervention, nurses described situations in which they were unable to secure sufficient time to engage with patients because of their competing clinical responsibilities. Furthermore, because the outpatient clinic is not a specialized transition service, nurses described practical difficulties in continuously integrating the guideline into everyday practice.

*“Because our clinic is not a specialized transition service, we cannot bill for these activities. Ideally, having a dedicated clinic where patients and families could be seen regularly would make continuous use of the guideline much more feasible.”* (Nurse C).

##### 3.2.3.3 Category 3. Team-based support enabled by a shared guideline, alongside emerging role ambiguity

The guideline was recognized as providing a shared framework that facilitated team-based transition support. Its implementation helped raise collective awareness within a small core team and enabled members to articulate common care directions. Nurses described having experienced nurses who could independently utilize the guideline, along with cooperation from physicians, which supported its use in practice.

For example, the nurses reported that the guideline served as a mediating tool, making it easier to share care perspectives within the team and discuss role allocation in collaboration with physicians. The nurses described how increased dialog among team members helped them view transition support as a shared team responsibility rather than an individual effort.

*“It would have been difficult without physicians’ cooperation, especially in securing time. By reviewing the assessment sheet, nurses could identify issues in advance and know what topics should be discussed with physicians. As a result, we had more opportunities to talk with physicians and patients, and this guideline created opportunities for staff members to share information, which I think was very beneficial.”* (Nurse A).

However, the nurses anticipated that if the number of staff members involved increased, maintaining role clarity would become more difficult. They expressed concerns that primary accountability for transition-related interventions might become less clear and that adherence to the guideline could become more variable. From the perspective of team leaders, the burden associated with coordinating team building and ongoing management was also described as a concern regarding the sustained use of the guideline over time.

*“When more people become involved in using the guideline, it can feel as though individual responsibility becomes diluted, making collaboration within the team more difficult.”* (Nurse C).

##### 3.2.3.4 Category 4. Interdepartmental collaboration initiated by broadened staff awareness constrained by limited organizational uptake

The nurses stated that the use of the guideline broadened their perspectives and, in some cases, was accompanied by moves toward interdepartmental collaboration.

For example, they described considering referral for nutrition counseling in anticipation of diet-related issues and being mindful of the need for coordination with ward staff when hospitalization was anticipated.

*“At that timing, before entering high school, when school lunches would no longer be available, we thought, ‘Maybe we should arrange a nutrition consultation at this point.’ It gave us an opportunity to step in proactively.”* (Nurse B).

By contrast, because the guideline was not positioned as a shared organizational tool, cross-unit information sharing and collaboration remained limited. Nurses reported cases in which they learned only afterward that the AYA they had been supporting in the outpatient setting had been urgently admitted at night. They described that, in such situations, changes in clinical condition were not always recognized as opportunities for timely transition-related intervention. Nurses stated that support within outpatient care alone was sometimes insufficient and raised concerns regarding information sharing and coordination between outpatient and ward settings.

*“Although we used a paper-based guideline, a remaining challenge is how to hand over the assessment findings when patients are admitted. If the guideline could be incorporated into the electronic medical record and used across the organization, it would be much easier to use and would become something routinely expected. For effective utilization, regular and mandatory assessment would need to become part of usual practice.”* (Nurse C).

Across all categories, nurses consistently described the guideline as a structured framework that supported assessment, communication, and transition-related support in routine outpatient care. However, sustained implementation was perceived to depend on contextual factors, including time availability, workload, role clarity, and organizational integration.

#### 3.2.4 Changes assessed using the Am I ON TRAC questionnaire

A comparison of the pre- and post-intervention scores on the Am I ON TRAC for Adult Care Questionnaire (validated Japanese version) revealed no significant differences for either the AYAs or their parents (Table 3). Owing to missing follow-up data, descriptive comparisons were available for four cases (Cases Nos. 1, 2, 5, and 6).

**Table 3:** Descriptive changes in transition readiness scores before and after the intervention (AYAs and their parents).

Domain	AYA Pre (mean ± SD)	AYA Post (mean ± SD)	Parent Pre (mean ± SD)	Parent Post (mean ± SD)
Knowledge score (total)	39.5 ± 4.6 (n = 6)	38.3 ± 5.5 (n = 4)	36.3 ± 5.0 (n = 6)	38.3 ± 5.8 (n = 4)
Behavior score (total)	4.3 ± 1.7 (n = 6)	2.5 ± 2.1 (n = 4)	2.8 ± 0.7 (n = 6)	2.5 ± 0.9 (n = 4)

*Values are presented as mean ± SD. Follow-up questionnaire data were available for four AYAs and their parents.

Descriptively, knowledge scores remained relatively stable among the AYAs over the 12-month period, whereas parental knowledge scores showed a slight increase. In contrast, behavior scores descriptively decreased among both AYAs and parents. However, given the small sample size and the use of the questionnaire as a reference indicator, these quantitative patterns should be interpreted cautiously. Table 3 presents the descriptive score patterns alongside the qualitative findings.

## 4 Discussion

A key contribution of this study lies in bridging theory and practice by translating a previously developed responsibility transition process model into a practical care guideline and examining its application in routine clinical settings. Through clinical implementation, this study clarified both the practical value of the guideline and the contextual challenges influencing its sustained use. The findings demonstrated that the guideline was feasible in routine pediatric care and provided nurses with a shared frame of reference when engaging with AYAs and their parents.

Phase 2 expert feedback informed the refinement of the guideline, and Phase 3 implementation data shaped the interpretation of its usefulness and challenges in practice. In this mixed-methods study, qualitative findings provided essential context for interpreting the quantitative evaluation. These findings underscore the limitations of relying solely on a single quantitative indicator to evaluate transition-related interventions and highlight the importance of practice-based knowledge that becomes visible through clinical implementation.

### 4.1 Usefulness of the care guideline and its influence on AYAs and their parents

Findings regarding the use of the care guideline revealed that nurses often experienced difficulty in determining appropriate directions for intervention when caring for AYAs with biliary atresia surviving with their native livers. This difficulty arose from several contextual factors, including long-standing parent–child relationships established since early childhood, the ethically sensitive nature of discussing the possibility of future living-donor liver transplantation, and the fact that nursing involvement in outpatient clinics and pediatric wards was often intermittent or limited to relatively short periods. Under these clinical circumstances, nursing care tended to prioritize immediate physical concerns, such as monitoring examination results and managing cholangitis, leaving limited opportunities to address longer-term issues related to responsibility transition and self-management. An important strength of the present care guideline lies in its ability to reframe everyday illness management within the context of parent–child relationships and to provide nurses with a systematic perspective for assessing the perceptions and behaviors of both AYAs and their parents.

Although all participants in the present study were aged 15 years or older, nurses recognized that the complexity of long-established parent–child relationships and the prospect of future liver transplantation sometimes made it difficult to move beyond immediate adjustments, resulting in sensitive issues being deferred. These findings suggest that some challenges related to parent–child relationships and the transfer of responsibility may emerge before mid-adolescence and become increasingly difficult to address once interactional patterns have become established. Got Transition’s Six Core Elements of Health Care Transition recommend initiating the transition process in early adolescence (approximately 12–14 years of age), followed by ongoing assessment of transition readiness and individualized transition planning throughout adolescence.^22^ More broadly, established transition guidance emphasizes supporting the gradual transfer of responsibility for care according to each adolescent’s developmental stage and readiness.¹ The original process model^10^ provided a conceptual framework for visualizing stage-specific interventions that considered both developmental stage and relational dynamics as care shifted from parent-led illness management to AYA-centered management of illness and daily life. The present care guideline operationalizes this framework into a format that is accessible and usable in routine nursing practice. Its principal strength lies in translating a theoretical model into a practical tool that supports the focus and goal setting of nursing interventions.

In particular, the possibility of future living-donor liver transplantation represents a unique and enduring concern for AYAs with biliary atresia surviving with their native livers and their parents. In the present study, nurses likewise recognized these issues as barriers to deepening intervention. Discussions concerning living-donor liver transplantation are ethically sensitive and may discourage proactive dialog. Furthermore, because uncertainty remains regarding the timing and indications for transplantation in biliary atresia,^23^, ^24^ it is important to assess how AYAs and their parents perceive and live with this uncertainty. Through refinement based on expert feedback and clinical implementation, the present guideline enables nurses to attend not only to concerns already expressed by AYAs and their parents but also to latent issues that may emerge in the future, while facilitating the selection of realistic goals appropriate to each individual’s current stage of responsibility transition. Furthermore, introducing the guideline at an earlier developmental stage may facilitate gradual, developmentally appropriate discussions regarding the potential need for future transplantation before it becomes an imminent clinical concern. Future studies are warranted to examine the feasibility and effectiveness of implementing the guideline among younger adolescents.

The absence of significant changes in Am I ON TRAC scores did not necessarily indicate that the intervention lacked usefulness. Transition readiness reflects a developmental and relational process that evolves gradually over time, and self-report measures focusing primarily on knowledge and behavior may be insufficiently sensitive to detect short-term or subtle changes in parent–child relationships and the transfer of responsibility. Participation in the intervention may also have encouraged some AYAs to reflect more critically on their self-management practices, thereby influencing their self-evaluation of behaviors. However, given the very small number of participants with complete follow-up data, it remains unclear whether these patterns reflected increased self-awareness, measurement limitations, unintended consequences of the intervention, or simply random variation. Moreover, the present intervention targeted not only individual knowledge and behavior but also relational processes, including parent–child dynamics and the gradual transfer of caregiving responsibility. Considering the 12-month implementation period, the limited sample size, and the developmental nature of responsibility transition, measurable changes in overall transition readiness scores may not have emerged within the study timeframe. Recent reviews have highlighted substantial variability in outcome measures used to evaluate transition interventions, as well as the limitations of relying on a single indicator.^4^, ^25^ Developing evaluation approaches that better align with the relational focus of the present guideline therefore remains an important challenge for future research. Further qualitative inquiry involving AYAs themselves is also needed to deepen understanding of their intervention experiences and changes in self-awareness.

Previous transition research has emphasized parental involvement primarily as a factor facilitating transition, whereas its potential to generate tension and difficulty has received considerably less attention. In the Japanese context of biliary atresia, where parents themselves may become future living donors, the present study demonstrated the ambivalent nature of parental involvement as both a source of support and a potential barrier to responsibility transition. This finding extends the existing transition literature across other chronic conditions. Previous studies suggest that parental involvement adapted to the adolescent’s developmental stage facilitates transition, whereas excessive parental control during adolescence and young adulthood may impede it.^26^ The present study advances this understanding by situating these general observations within the highly relational context of potential living-donor liver transplantation and illustrating the dual nature of parental involvement in responsibility transition.

### 4.2 Usefulness and challenges revealed through clinical implementation

A notable feature of this study is that the care guideline was not limited to use as an evaluative tool but was actively referred to and used by nurses in actual outpatient clinical settings. Through the use of the guideline, nurses expanded their focus beyond conventional assessments of physical condition and test results to include AYAs’ reflections on their daily lives with illness and the nature of parental involvement. For example, nurses described that conducting assessments while referring to the guideline during outpatient visits created opportunities to share previously unarticulated strategies and difficulties in daily life, as well as the roles assumed by parents in disease management. These shared reflections facilitated the alignment of perspectives on living with illness among AYAs, parents, and nurses. In this way, the guideline functioned as a concrete reference framework that clarified “what to ask” and “where to focus” in nursing practice.

Qualitative analysis further revealed that the usefulness and challenges arising from the clinical implementation of the care guideline did not emerge independently but as paired, interrelated concepts. While use of the guideline prompted AYAs to reflect on their daily lives and adjust their illness management, uncertainty regarding future liver transplantation and the complexity of parent–child relationships were simultaneously recognized as barriers to more proactive and future-oriented discussions. Similarly, although nurses reported increased awareness of transition support, limited time and workload in outpatient settings hindered sustained use of the guideline.

The guideline promoted team-based care by providing a shared perspective, however, involvement of a larger number of staff members sometimes resulted in role ambiguity. Collaboration with other departments was occasionally facilitated; however, when dissemination across the organization was insufficient, opportunities to maintain continuity of transition support during acute changes in clinical status remained limited. These findings suggest that increased staff awareness may serve as a catalyst for new interdisciplinary collaboration. This interpretation is consistent with previous literature indicating that insufficient staff knowledge and organizational preparedness can impede healthcare transition, whereas increasing staff awareness may facilitate interdisciplinary collaboration and coordinated transition support.^1^ At the same time, these challenges appear to reflect organizational and contextual factors within the implementation process rather than deficiencies in the guideline itself.

Overall, the findings indicate that sustained use of the guideline was contingent on contextual factors such as time and workload, role clarity within teams, and cross-departmental information sharing. Nurses’ accounts suggested that enabling conditions and constraints tended to coexist across the levels of AYAs and parents, clinicians, teams, and organizations.

Therefore, beyond effectiveness alone, implementation outcomes such as feasibility, usability, and sustainability warrant consideration. The challenges identified in this study are consistent with prior findings in implementation science and guideline research, emphasizing the importance of fit within the clinical context and user-centered design.^11^, ^27^ However, the contribution of this study extends beyond providing theoretical support for these concepts. By examining actual outpatient clinical settings, this study illustrates how and under what conditions the guideline was used, as well as the conditions under which its use became difficult to sustain. These findings indicate that the value and limitations of a care guideline become visible not at the point of development, but through the process of its use in everyday clinical practice.

### 4.3 Practical suggestions for supporting sustained use

Consistent with the qualitative categories described above, several practical suggestions are offered to support the sustained use of the guideline in routine outpatient care:

• Explicitly sharing goals with AYAs and their parents,
• Specifications of intervention timing aligned with the developmental stages,
• Simplification of assessment items to fit routine workflows, and
• Alignment of the guideline with existing hospital systems and documentation processes.

Accordingly, this study is meaningful in that it moves beyond evaluating a care guideline as simply “good” or “effective” and demonstrates its potential to be connected to implementation outcomes that reflect whether it is “usable” and “used.” Through the introduction of the guideline, emerging changes in nursing practice and concrete factors that hinder sustained use were identified, providing practical insights for future refinement and broader implementation.

Although the relational dynamics observed in this study are shaped by the Japanese context—particularly the high prevalence of parent-to-child living-donor liver transplantation—the underlying process of negotiating responsibility between parents and adolescents is not unique to Japan. Similar tensions between autonomy and parental involvement have been reported across chronic conditions and healthcare systems, particularly during health care transition. However, cultural norms may influence how these processes unfold in practice. ^28^^,^^29^ Therefore, contextual adaptation is necessary when implementing the guideline in different settings.

### 4.4 Limitations and future directions

This study had several limitations. First, the small sample size and single-site design limit the generalizability of our findings. Second, there was a substantial time interval between the original qualitative studies that informed the guideline, its clinical implementation, and the present publication. Although the guideline focuses on relational and developmental processes that remain relevant to transition support, this time lag should be considered when interpreting the findings.

Third, the “Am I ON TRAC” survey may not have fully captured the relational and process-oriented changes targeted by the intervention. In addition, because this study focused on nurses’ perceptions of guideline use, firsthand accounts from AYAs and their parents were not collected. Future studies incorporating interviews or other qualitative approaches with AYAs and their parents would provide a more comprehensive understanding of the experiences and perceived impact of the guideline. As an inherent constraint of this mixed-methods study, the small number of participants with complete follow-up data limited the ability to detect quantitative changes. Therefore, the quantitative findings should be interpreted descriptively and considered alongside the qualitative findings, which provide important contextual information regarding implementation outcomes.

Fourth, because all participating AYAs were aged 15 years or older, the applicability of the guideline to younger adolescents remains unclear. Furthermore, long-term outcomes were not evaluated. In addition, when presenting the guideline in an international context, emphasis was placed on clarity and usability. Accordingly, the appendix presents the theoretically grounded operational core components of the guideline rather than the full detailed assessment checklist. The complete version, including the detailed assessment items, remains available in the original Japanese version and may be provided upon request.

Future research should include larger samples and multisite designs to enhance the generalizability of the findings and should examine the feasibility of introducing guideline-based support at earlier stages of adolescence. Although this guideline was developed in the context of biliary atresia, the relational process of responsibility transition between parents and AYAs may be relevant to other childhood-onset chronic conditions. Further studies are required to examine contextual adaptations across diagnoses and healthcare systems. In addition, the development of evaluation measures capable of capturing relational processes, responsibility transitions, and their associations with long-term transition outcomes is warranted.

## 5 Conclusion

In this study, a care guideline for AYAs with biliary atresia surviving with their native livers and their parents was developed and refined through expert review, and its usefulness and implementation challenges were examined in clinical practice.

This study extends previous work on responsibility transition by translating a relational process model into a clinically implementable guideline and demonstrating its feasibility within routine pediatric outpatient care. Rather than evaluating the guideline solely in terms of effectiveness, the findings underscore the importance of examining implementation outcomes, including usability, adaptability, and sustained use in real-world settings. Although the guideline was feasible in routine pediatric practice, contextual factors such as time availability, organizational integration, and interprofessional collaboration influenced its continued use.

The relational processes underlying responsibility transition may also be relevant for AYAs with other childhood-onset chronic conditions. Future studies should examine the transferability and adaptation of this approach across diverse clinical contexts to support self-management and responsibility transition among AYAs and their families.

## CRediT authorship contribution statement

**Nobue Nakamura:** Writing – review & editing, Validation, Supervision, Conceptualization. **Hiroko Watayo:** Writing – review & editing, Validation, Supervision. **Katsuhiro Hiratsuka:** Writing – original draft, Visualization, Supervision, Resources, Project administration, Methodology, Investigation, Funding acquisition, Formal analysis, Data curation, Conceptualization.

## Ethical considerations

This study was conducted in accordance with the Declaration of Helsinki and was approved by the Ethics Committee of the Graduate School of Nursing, Chiba University, Japan (No. 30–17); the Ethics Committee for Research Involving Human Subjects, Sophia University (No. 2020–91); and the Ethics Committee of the Faculty of Medicine, Juntendo University (No. E21–0314).

## Statement for studies in humans

This study involved human participants and was conducted in accordance with the Declaration of Helsinki. The study protocol was approved by the institutional review board (details blinded for review). Written informed consent was obtained from all participants (and parents/guardians where applicable), and participants’ privacy rights were observed.

## Funding

This work was supported by JSPS KAKENHI [grant numbers JP17H07104, JP20K19188]. The funding body had no role in the study design; data collection, analysis, or interpretation; manuscript preparation; or the decision to submit the article for publication.

## Declaration of Competing Interest

The authors declare that they have no known competing financial interests or personal relationships that could have appeared to influence the work reported in this paper.

## Data Availability

The data that has been used is confidential.
