# Supplementary material for: Development and clinical implementation of a care guideline to support self-management and responsibility transition in adolescents and young adults with biliary atresia
Source: Health Care Transit. 2026 Jul 28;4:100150. doi: 10.1016/j.hctj.2026.100150 (PMC13450250; doi:10.1016/j.hctj.2026.100150)
Supplement: Supplementary file 1 [file mmc1.docx]

**Appendix 1. Core Clinical Version of the Care Guideline**

This appendix presents the core operational components of the care guideline, including its theoretical foundation, procedures for use, phase-specific achievement goals, and support strategies. These components are intended to provide sufficient information for understanding, implementing, and adapting the guideline in routine clinical practice.

Although the original Japanese version contains more detailed assessment checklists, the essential elements necessary for clinical application are fully described in this appendix.

**Care Guideline to Support Self-management and Responsibility Transition**

**in Adolescents and Young Adults with Biliary Atresia**

**1	Conceptual Background and Purpose**

**1.1	Background**

Biliary atresia is a neonatal-onset cholestatic disease that requires lifelong medical follow-up owing to progressive complications. Although long-term survival has improved, adolescents and young adults (AYAs) surviving with their native livers continue to face challenges in self-management and uncertainty regarding potential living-donor liver transplantation.

Developmental changes intersect with disease-related demands during adolescence and young adulthood. Parents, particularly as potential future living donors, often remain highly involved in treatment decisions and illness management. This unique context may complicate the gradual transition of responsibility for daily life management, illness management, and health-related decision-making from parents to AYAs.

**1.2	Purpose of this Guideline**

This care guideline aims to:

1. Support AYAs in organizing and sustaining self-management.
2. Enhance psychological and practical preparedness for living with the possibility of future living-donor liver transplantation.
3. Provide healthcare professionals with a structured framework aligned with the Process Model for Transitioning Care Responsibility.

This guideline consists of three core components:

- An assessment framework based on four domains;
- Phase-specific achievement goals; and
- Phase-specific support strategies designed to facilitate responsibility transition.

**1.3	Theoretical Foundation**

This guideline is grounded in the Process Model for Transitioning Care Responsibility, which identifies four phases:

1. Parent-led recuperation: Parents assume primary responsibility for illness management, while AYAs have limited illness ownership and often follow parent-led care.
2. A vicious cycle of control and rebellion: AYAs’ autonomy begins to emerge, but parental control may intensify, leading to tension, resistance, or avoidance around illness management.
3. Passing the axis of responsibility: Responsibility begins to shift from parents to AYAs through gradual delegation, shared planning, and changes in both AYA and parent roles.
4. Aligning the parent–child rhythm to create a patient-centered life: AYAs take the lead in organizing daily life with illness, while parents provide supportive rather than controlling involvement.

In this guideline, responsibility transition is defined as the gradual shift in responsibility for daily life management, illness management, and health-related decision-making from parents to AYAs.

Responsibility transition is understood as a dynamic and context-sensitive process through which AYAs and parents mutually adapt through communication, negotiation, and reflection. Progression is nonlinear, and movement between phases may occur in either direction according to developmental, relational, and clinical circumstances.

**Figure S1. Process model for the transition of daily responsibility from parents to adolescents and young adults with biliary atresia**

Joint display of the process model and prior findings. The ovals indicate the relationship between the process phases and key categories derived from two prior studies, functioning as a joint display that integrates the perspectives of AYAs and their parents. Note: This figure illustrates the transition of daily responsibilities from parents to AYAs with biliary atresia who survive with their native livers. The model is based on previous qualitative studies (Hiratsuka, 2021; Hiratsuka et al., 2021). The horizontal axis in the center (blue area) represents four inductively derived phases: “parent-led recuperation,” “vicious cycle of control and rebellion,” “passing the axis of responsibility,” and “aligning the parent–child rhythm to create a patient-centered life.” The arrows indicate the direction of change and influence over time. Ovals represent the key conceptual categories identified in previous AYA-focused- and parent-focused studies. These categories are visually mapped onto the four process phases to demonstrate how the original findings informed the current model. Thus, the ovals function as part of a joint display, integrating insights from both primary and secondary analyses and demonstrating how perspectives from AYAs and parents interweave and evolve throughout the transition process.

**2	How to Use this Guideline**

**2.1	General Principles of Use**

**2.1.1	Core Principles**

- This guideline is not intended to be completed exhaustively during a single encounter.
- Assessment may proceed progressively based on the available clinical information.
- Missing information may itself be clinically meaningful.
- Items related to liver transplantation should be approached with clinical and ethical sensitivity.
- Reassessment and reclassification are expected.

**2.1.2	Target AYAs and Parents**

This guideline applies primarily to:

- AYAs aged approximately 12–29 years surviving with their native livers.
- Individuals not currently experiencing decompensated cirrhosis requiring immediate transplantation.
- Parents who have primarily managed treatment and accompanied outpatient visits.

**2.1.3	Intended Users**

The guideline was primarily developed for outpatient nurses, although it may also inform the practice of other healthcare professionals involved in the longitudinal care of AYAs with biliary atresia and their families.

The guideline is intended for:

- Registered nurses;
- Certified nurses or nurse specialists;
- Transplant coordinators; and
- Healthcare professionals involved in pediatric, hepatobiliary, or transplant care.

**2.1.4	Timing of Use and Location**

The guideline is intended for repeated use during routine outpatient encounters and may be revisited as AYAs and families progress through different phases of responsibility transition.

This guideline may be applied:

- During routine outpatient visits;
- During hospitalization, when opportunities arise to address issues related to responsibility transition; and
- During face-to-face encounters in outpatient or inpatient settings, according to clinical need.

Given the limited opportunities for contact in clinically stable cases, users of this guideline are encouraged to incorporate structured assessments whenever meaningful opportunities for interaction arise.

**2.2	Steps of Use**

The following steps are designed to integrate guideline use into routine outpatient workflows without substantially increasing the workload.

**2.2.1	Step 1: Rapid Orientation (5–10 minutes)**

Before proceeding to structured assessment, conduct a brief orientation to understand the current clinical and relational context.

Consider the following:

- Is the AYA medically stable and not currently under consideration for urgent transplantation?
- Who is primarily managing daily life and treatment decisions?
- What characterizes the current parent–child interaction?
- Who usually communicates with healthcare professionals?
- Who manages clinic appointments?
- Has the possibility of future transplantation been discussed?
- Are concerns about future transplantation evident?

Based on this rapid orientation, identify the phase that appears most consistent with the current situation as an initial clinical impression.

If the phase is reasonably clear, proceed **to Step 3** to confirm phase identification and select goals.

If uncertainty remains, proceed **to Step 2** for structured assessment.

**2.2.2	Step 2: Structured Assessment**

The assessment consists of four domains:

Assessment A. Physical status, disease severity, transplantation-related conditions, and daily life circumstances.

Assessment B. Illness ownership, meaning-making, self-management experiences, lifestyle adjustment, and uncertainty regarding the future.

Assessment C. Parental delegation of responsibility, perceptions of illness, and adaptation to changing caregiving roles.

Assessment D. Patterns of parent–AYA interaction and phase-related relational characteristics.

Assessment may be conducted non-linearly depending on the clinical context.

Examples of detailed assessment items included in the original Japanese version encompass medication management, appointment scheduling, lifestyle adjustments, perceptions regarding future transplantation, parental delegation of responsibility, and patterns of parent–AYA communication.

**2.2.3	Step 3: Phase Identification**

Using the findings from Domains A–D, determine the phase that most closely reflects the current AYA–parent dyadic pattern. These four domains collectively guide phase identification and the subsequent selection of phase-specific achievement goals and support strategies.

Phase identification remains provisional and may be revised during reassessment.

**Box S1. Operational Logic for Phase Identification**

Characterized by low AYA autonomy and high parental control

**→ Phase 1: Parent-led recuperation**

Characterized by emerging AYA autonomy and escalating parental control

**→ Phase 2: Vicious cycle of control and rebellion**

Characterized by increasing AYA autonomy and gradual parental delegation

**→ Phase 3: Passing the axis of responsibility**

Characterized by stable AYA autonomy and mutual parental adaptation

**→ Phase 4: Aligning the parent–child rhythm to create a patient-centered life**

Phases are determined based on the dynamic configuration of AYA autonomy and parental involvement. The phase classification is provisional and may change over time.

**2.2.4	Step 4: Selection of Phase-Specific Goals**

Select goals aligned with the identified phase. If the goals of an earlier phase remain partially unmet, the strategies from that phase may be reintroduced.

**2.2.5	Step 5: Implementation of Targeted Support Strategies**

Guideline-based support strategies include:

- Facilitated reflection and meaning-making;
- Psychoeducational support;
- Behavioral adjustment support;
- Structured dialogue between AYAs and parents; and
- Support for coping with transplantation-related uncertainty.

Interventions should be individualized according to the AYA’s developmental readiness, clinical status, family dynamics, and evolving concerns regarding future transplantation.

**2.2.6	Step 6: Reassessment**

Reassessment should consider changes in clinical condition, developmental tasks, parent–AYA interactions, and emerging future concerns. Movement between phases is expected.

**Box S2. Iterative Cycle of Guideline-Based Care**

Assessment → Goal Setting → Intervention → Reflection → Phase Reclassification

The process proceeds from assessment to goal setting, intervention, reflection, and phase reclassification. Reassessment and adjustment are expected as clinical and relational conditions evolve.

**3	Phase-based Achievement Goals and Support Strategies**

Phase-based achievement goals and support strategies should be selected according to the phase identified in the previous step. Because phase identification is provisional, goals and strategies may overlap between adjacent phases and should be adapted to individual contexts.

**Box S3. Operational Principles for Goal Selection**

Goal selection should be guided primarily by the identified phase; however, goals from adjacent phases may also be incorporated according to developmental readiness, clinical circumstances, and family needs.

The following tables provide phase-based clinical guidance for routine practice, outlining key focus areas, achievement goals, and support strategies.

**Phase 1: Parent-led recuperation**

| Domain | Clinical Focus |
| --- | --- |
| AYA | Limited illness ownership; low autonomy |
| Parent | High control; protection-oriented |
| Achievement Goals | Promote awareness of illness experiences, emerging questions, and meaning-making. |
| Support Strategies | - Encourage expression of questions and illness-related concerns;  - Provide age-appropriate explanations;  - Reinforce small acts of self-management;  - Support recognition of emerging autonomy; and  - Facilitate meaning-making regarding uncertainty and illness experiences. |

**Phase 2: Vicious cycle of control and rebellion**

| Domain | Clinical Focus |
| --- | --- |
| AYA | Emerging autonomy; resistance or avoidance |
| Parent | Escalating control; anxiety-driven involvement |
| Achievement Goals | Reduce escalation of parent–AYA conflict and support identity integration. |
| Support Strategies | - Provide opportunities for individual conversations;  - Validate emerging autonomy;  - Reframe conflict as part of normative development;  - Facilitate structured parent–AYA dialogue; and  - Explore selective disclosure and social participation. |

**Phase 3: Passing the axis of responsibility**

| Domain | Clinical Focus |
| --- | --- |
| AYA | Increasing autonomy; future orientation |
| Parent | Gradual delegation; ambivalence |
| Achievement Goals | Support the gradual transfer of responsibility and future-oriented self-management. |
| Support Strategies | - Collaborative care planning;  - Review lifestyle–treatment balance;  - Facilitate age-appropriate discussion regarding future transplantation;  - Support coping with uncertainty;  - Promote social role attainment; and  - Redefine the parent role as a consultant. |

**Phase 4: Aligning the parent–child rhythm to create a patient-centered life**

| Domain | Clinical Focus |
| --- | --- |
| AYA | Stable self-management; adaptive coping |
| Parent | Supportive presence; reduced control |
| Achievement Goals | Sustain autonomy, adaptive self-management, and preparedness for future uncertainty. |
| Support Strategies | - Periodic reflective review;  - Reinforce competence;  - Maintain open discussion regarding uncertainty;  - Support preparedness for potential transplantation;  - Facilitate family communication; and  - Maintain access to ongoing professional support. |

**Box S4. Principles for Supporting Uncertainty**

Supporting AYAs and parents to tolerate uncertainty and adopt a probabilistic understanding of uncertainty related to disease progression, future complications, and the possibility of transplantation may facilitate responsibility transition and long-term self-management.
